# Study of the correlation between serum ferritin levels and the aggregation of metabolic disorders in non-diabetic elderly patients

**DOI:** 10.3892/etm.2014.1668

**Published:** 2014-04-07

**Authors:** BIQIANG LI, WEI LIN, NAN LIN, XIAOWEN DONG, LIBIN LIU

**Affiliations:** 1 Department of Cadre Ward, Fujian Medical University Union Hospital, Fuzhou, Fujian 350001, P.R. China; 2 Department of Endocrinology, Fujian Medical University Union Hospital, Fuzhou, Fujian 350001, P.R. China

**Keywords:** serum ferritin, metabolic disorder, diabetes, insulin

## Abstract

The present study aimed to explore the correlation between serum ferritin (SF) levels and the aggregation of metabolic disorders in non-diabetic elderly patients. A total of 2,600 patients were enrolled in the study. Various parameters, including blood pressure (BP), height, weight, lipid profiles, blood glucose (BG), body mass index (BMI), fasting insulin (FINS), serum uric acid (SUA), the urinary albumin/creatinine ratio (UACR) and SF levels were measured. A homeostatic model was used to evaluate insulin resistance (HOMA-IR) and β-cell function (HOMA-β). The quantitative insulin sensitivity check index (QUICKI) and disposition index (DI) were calculated. The QUICKI and DI decreased significantly and other parameters increased significantly when the number of metabolic disorders increased. Patients with high triglycerides (TG), high total cholesterol (TC), high SUA and obesity demonstrated higher SF levels than those with normal TG, normal TC, normal SUA and normal weight, respectively (P<0.01). Male patients with metabolic disorders (high TG, high TC, high BP, high SUA and obesity) had higher SF levels than female patients with the corresponding disorders (P<0.01). BG, FINS, BMI, TC, TG, SUA, HOMA-IR and HOMA-β were positively correlated with SF, while DI and QUICKI were negatively correlated with SF (P<0.01). Stepwise regression analysis showed that HOMA-IR, BMI, TC, TG and SUA were risk factors for elevated SF levels. In conclusion, the SF levels in non-diabetic, elderly individuals with metabolic disorders may be significantly related to the clustering of the metabolic disorders. Dyslipidemia, obesity, disorders of purine metabolism and insulin resistance may be important risk factors for higher SF levels in the elderly.

## Introduction

Metabolic syndrome (MS) is composed of a group of irregularly aggregated metabolic components with clinical characteristics of hypertension, diabetes, abnormally regulated glucose, metabolic disturbance of blood lipids and obesity. A common pathological and physiological feature of MS is insulin resistance (1). The various components of MS are all important risk factors of cardiovascular disease (CVD) and they play a critical role in the pathogenesis and development of CVD. Once various abnormal metabolic components are increased or aggravated, the prevalence of CVD increases significantly (2,3).

Studies have revealed that the risk factors of CVD, including disorders of lipid metabolism, hypertension, damaged regulation of fasting blood glucose (BG) and obesity, are closely associated with serum ferritin (SF) levels (4–7). Compared with young individuals, older individuals are more prone to a variety of metabolic disorders, particularly hypertension, disorders of lipid metabolism, obesity, abnormal glucose metabolism and hyperuricemia. However, the pathogenesis of this remains to be investigated.

In the present study, the relationship between SF levels and the aggregation of metabolic disorders in non-diabetic elderly patients was investigated by analyzing data from the physical examinations of the elderly patients.

## Materials and methods

### Patients

A total of 2,600 elderly individuals who visited the Affiliated Union Hospital (Fuzhou, China) for physical examinations from March to December 2012 were investigated in the present study. All patients were more than 60 years old and the average age was 69.25±5.26 years. There were 1,500 males (57.69%) and 1,100 females (42.31%). Of these, there were 1,610 individuals with normal glucose tolerance, 890 individuals with abnormal glucose tolerance and 100 individuals with impaired fasting glucose. According to the exclusion criterion of the World Health Organization (WHO) in 1999, diabetic patients were excluded (4). In addition, patients with acute chronic inflammatory diseases, anemia, a recent blood transfusion and use of iron, malignant tumors, autoimmune or hereditary diseases, hyperthyroidism or alcoholism were excluded. For experiments involving human patients, approval was obtained from the institutional review committee of the Affiliated Union Hospital. Informed consent was provided from each patient according to the Declaration of Helsinki.

### Parameter examinations

All patients were asked to sit relaxed for at least 5 min. Right (upper) arm brachial arterial blood pressure (BP) was measured by a mercury column sphygmomanometer. BP was detected three times and an average BP was recorded and analyzed.

Body weight and height were measured, to an accuracy of 0.1 kg and 0.1 cm, respectively, using an adjusted weighing machine and a height measuring instrument with the help of a trained assistant. The body mass index (BMI) was calculated and the body fat content (BFC) was evaluated using the following formulae: Male BFC = 1.2 × BMI + 0.23 × age − 16.2; and Female BFC = 1.2 × BMI + 0.3 × age − 5.4.

All patients were forbidden to drink for one day prior to the physical examination and were fasted for 10–12 h. At 7:00–8:00 a.m. on the day of physical examination, blood was drawn from a vein in the upper arm and the blood serum was separated. Glucose level was determined using the glucose oxidase method and total cholesterol (TC), triglycerides (TG), uric acid and urinary creatinine were examined using enzymatic methods. Levels of insulin were measured by the electrochemical immunoassay method using an electrochemical immunoassay kit (Roche Diagnostics, Basel, Switzerland). Urinary albumin was measured by immunoturbidimetry using a microalbumin test kit (Sigma, St. Louis, MO, USA). SF protein levels in the serum were measured by a radioimmunoassay method according to the manufacturer's instructions (Sigma). The ferritin protein was detected by radioimmunoassay method and ferritin mRNA was measured by the PCR method.

### Calculation of insulin resistance and pancreatic β-cell function indices

The homeostatic model assessment of insulin resistance (HOMA-IR) and homeostatic model assessment of pancreatic β-cell function (HOMA-β) indices were calculated using the following formulae: HOMA-IR = fasting insulin (FINS; Ins0) × fasting blood glucose (FPG)/22.5; and HOMA-β = 20 × Ins0/(FPG - 3.5).

The following were also calculated: Quantitative insulin sensitivity check index (QUICKI) = 1/(log fasting BG + log FINS); insulin disposition index (DI) = HOMA-β/HOMA-IR.

### Grouping of subjects

According to the diagnostic criteria for MS, suggested by the diabetes branch of the Chinese Medical Association, the patients were divided into the following groups: no metabolic disorders (MS0 group, n=1,150), one metabolic disorder (MS1 group, n=1,250), two metabolic disorders (MS2 group, n=150) and at least three metabolic disorders (MS3 group, n=50).

Based on a normal HOMA-IR value of 1.85, when the value was ≥2.15 this was defined as insulin resistance. Based on a normal HOMA-β value of 50.91, when the value was >141.79 this was defined as insulin secretion dysfunction. According to these criteria, the patients in the present study with normal glucose tolerance were divided into the following groups: Insulin sensitivity + insulin secretion dysfunction (SF1 group, n=431), insulin sensitivity + normal insulin secretion (SF2 group, n=751), insulin resistance + insulin secretion dysfunction (SF3 group, n=15), and insulin resistance + normal insulin secretion (SF4 group, n=436).

### Statistical analysis

All measured parameters were calculated and expressed as mean ± standard deviation. Any non-normally distributed data were transformed by a natural logarithm. A χ^2^ test was employed when comparing numerical data. Variance was analyzed using a one-way analysis of variance (ANOVA) and a Bonferroni correction with SPSS statistical software, version 15.0 (SPSS Inc., Chicago, IL, USA). Spearman correlation analysis and stepwise multiple linear regression analysis were used to analyze the correlation between SF and the metabolic indices. P<0.05 was considered to indicate a significant difference. Data from males and females were analyzed separately.

In the current study, the prevalence of diabetes in patients was explored and the data stratified according to age, gender, urban and rural living, and economic development level. The sample size met the accuracy requirements needed to carry out the complex survey. All calculations were weighted to represent the total Chinese population with ages of 20 years or older according to the nationwide census results of 2006. A χ^2^ test was employed when comparing prevalence of diabetes between groups. Continuous variables were compared using the general linear model after adjustments for age and BMI. Abnormally distributed continuous variables were compared after they were subjected to logarithmic transformation. Effects of geographic zoning, lifestyle and metabolic components on the prevalence of diabetes (odds ratio) were analyzed by multivariate logistic regression analysis. A backward selection method was used to select significant risk factors for the final risk model (P<0.05). Data are presented as a mean with 95% confidence interval or a median with the 25th to 75th percentile. SUDAAN 10.0 software (Research Triangle Institute, Research Triangle Park, NC, USA) was used to analyze data.

## Results

### Comparison of clinical parameters in different groups

As shown in Table I, as the numbers of metabolic disorders increased, the SF levels, BMI, systolic BP (SBP), diastolic BP (DBP), fasting BG, TC, TG, serum uric acid (SUA), FINS, BFC, HOMA-IR, HOMA-β and urinary albumin/creatinine ratio (UACR) increased gradually; whereas the QUICKI and DI decreased gradually.

### Comparison of concentrations of SF in patients with different characteristics

SF levels in patients with high TG (≥1.7 mmol/l, n=1,410) were higher than those in patients with normal TG (n=1,190; 5.3±0.6 vs. 5.2±0.5 μg/l, F=1.02, P=0.001). The SF levels in patients with high TC (≥5.7 mmol/l, n=1,235) were higher than those in patients with normal TC (n=1,365; 5.4±0.6 vs. 5.3±0.5 μg/l, F=1.72, P=0.001). Levels of SF in patients with hyperuricemia (SUA≥430 μmol/l, n=1,410) were higher than those in the group with normal SUA (n=1,190; 5.4±0.6 vs. 5.3±0.5 μg/l, F=1.42, P=0.001). Levels of SF in obese patients (BMI ≥25 kg/m^2^, n=810) were higher than those in patients of a healthy weight (n=1,790; 5.5±0.6 vs. 5.4±0.5 μg/l, F=3.12, P=0.001). Levels of SF in patients with hypertension (SBP ≥140 mm Hg or DBP ≥90 mm Hg, n=1,760) were higher than those with normal blood pressure (n=840; 5.3±0.5 vs. 5.2±0.4 μg/l, F=1.32, P=0.001). Levels of SF in males with normal glucose tolerance were higher than those in females with the same glucose tolerance level (5.3±0.5 vs. 5.2±0.6 μg/l, F=1.12, P=0.001). Levels of SF in males with metabolic abnormalities were higher than those in the corresponding group of females (5.5±0.5 vs. 5.4±0.6 μg/l, F=4.12, P=0.001). The levels of SF in males were also higher than those in the corresponding group of females for patients with high TC (5.6±0.6 vs. 5.5±0.5 μg/l, F=6.12, P=0.001), hypertension (5.5±0.6 vs. 5.4±0.5 μg/l, F=4.72, P=0.001), hyperuricemia (5.7±0.6 vs. 5.6±0.5 μg/l, F=3.82, P=0.001) and obesity (5.5±0.6 vs. 5.4±0.5 μg/l, F=3.72, P=0.001). No significant differences were identified between the groups SF1 and SF2 (5.2±0.6 vs. 5.2±0.5 μg/l, P=1.02), and between the groups SF3 and SF4 (5.7±0.7 vs. 5.7±0.6 μg/l, P=0.71) with respect to levels of SF. However, levels of SF in groups SF3 and SF4 were significantly higher than in groups SF1 and SF2 (P<0.01).

### SF mRNA and protein expression in different groups

**Figure 1:**
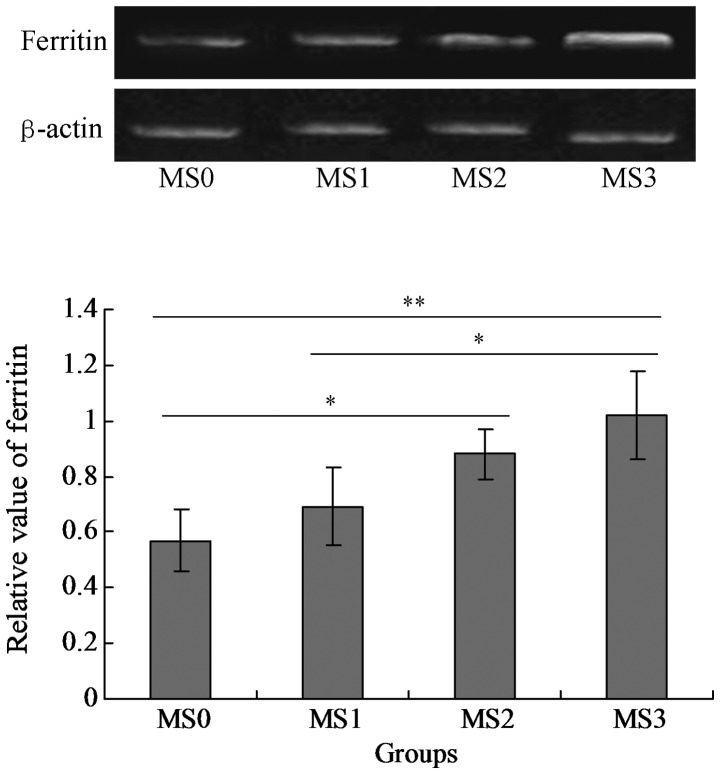
Ferritin mRNA expression in the groups. ^*^P<0.05, ^**^P<0.01 represent the differences between two groups.

**Figure 2:**
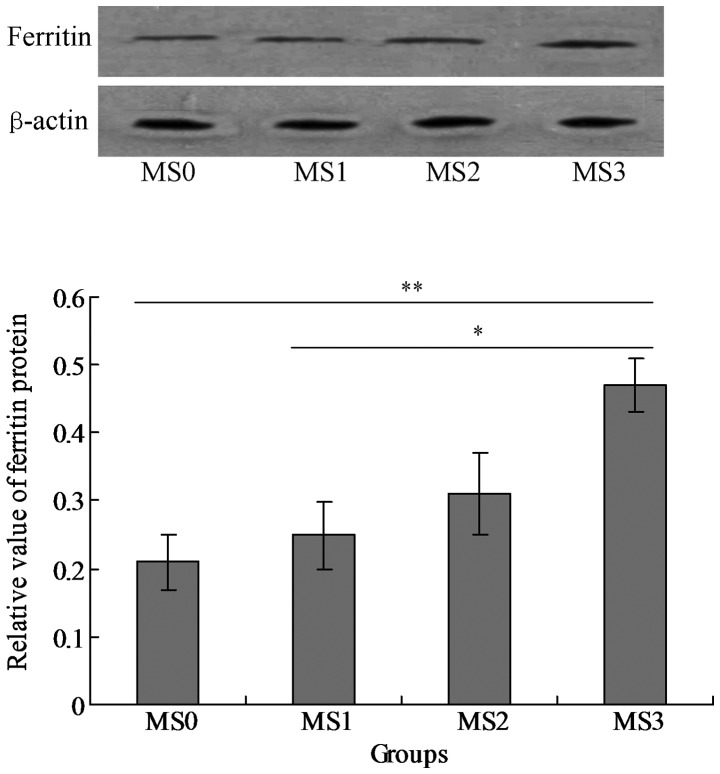
Ferritin protein expression in the groups. ^*^P<0.05, ^**^P<0.01 represent the differences between two groups.

The mRNA and protein expression levels of SF were detected in the blood serum samples. The results indicated that SF was expressed in the four groups MS0-MS4, however the level of SF (mRNA) expressed in group MS3 was significantly higher compared with that in the other three groups (P<0.05; Fig. 1). The SF protein was also expressed in all of the groups and the MS3 group expressed the highest level of SF protein among all the groups (P<0.05; Fig. 2).

### Results of Spearman correlation analysis

Results demonstrated that there were positive correlations between levels of SF and TG (r=0.10, P=0.001), TC (r=0.08, P=0.001), SUA (r=0.13, P=0.001), BMI (r=0.12, P=0.001), fasting BG (r=0.09, P=0.001), 2 h BG (r=0.11, P=0.001), FINS (r=0.17, P=0.001), HOMA-IR (r=0.19, P=0.001) and HOMA-β (r=0.12, P=0.001). However, SF levels were negatively correlated with QUICKI (r=−0.19, P=0.001) and DI (r=−0.10, P=0.001).

**Figure 3:**
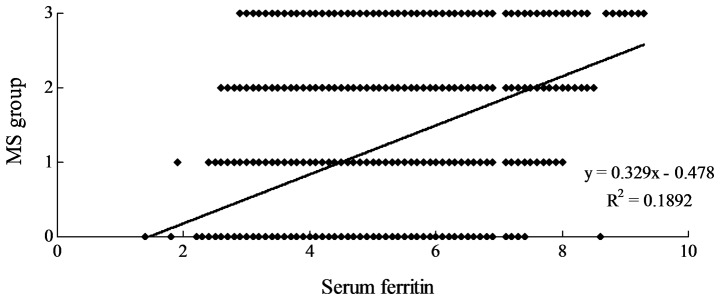
Correlation between ferritin expression in serum samples and the metabolic syndrome gradings.

Most notably, however, the SF level was positively correlated with the number of metabolic disorders (Fig. 3, r=0.3186, P<0.05).

### Multiple regression analysis of SF and the related metabolic indices

SF was used as a dependent variable and other related metabolic indices including BMI, SBP, DBP, fasting BG, TC, TG, SUA, BFC, FINS, HOMA-IR, UACR, FINS and DI were used as independent variables. Multiple regression analysis was conducted. The results in Table II demonstrate that BMI, TG, TC, SUA and the HOMA-IR are independent risk factors for increased levels of SF in non-diabetic elderly patients with disturbed metabolism.

## Discussion

The correlation between SF level and the aggregation of metabolic disorders in non-diabetic elderly patients was investigated in the present study. A total of 2,600 individuals were enrolled in the study. The blood pressure (BP), height, weight, lipid profiles, blood glucose (BG), body mass index (BMI), fasting insulin (FINS), serum uric acid (SUA), urinary album/creatinine ratio (UACR) and SF levels were measured. A homeostatic model was used to evaluate insulin resistance (HOMA-IR) and β-cell function (HOMA-β). The insulin sensitivity check index (QUICKI) and disposition index (DI) were calculated. Those with normal glucose tolerance were assigned to four groups (SF1, SF2, SF3 and SF4) according to the results of HOMA-IR and HOMA-β. The χ^2^ test and Spearman analysis were used for data comparison.

The level of SF, BMI, systolic blood pressure (SBP), diastolic blood pressure (DBP), SUA, total cholesterol (TC), triglyceride (TG), FINS, body fat content (BFC), HOMA-IR, and UACR significantly increased, while QUICKI and DI decreased, when the number of metabolic disorders increased. Patients with high TG, high TC, high SUA and obesity showed higher SF levels than those with normal TG, normal TC, normal SUA and normal weight, respectively (P<0.01). Male patients with metabolic disorders (high TG, high TC, high BP, high SUA and obesity) had higher SF levels than female patients with the same disorders (P<0.01). The 2 h BG, FINS, BMI, TC, TG, SUA, HOMA-IR and HOMA-β values were positively correlated with SF, while DI and QUICKI were negatively correlated with SF (P<0.01). Stepwise regression analysis showed that HOMA-IR, BMI, TC, TG and SUA were risk factors of SF.

The level of SF in the metabolic disorders of non-diabetic elderly individuals may be significantly related to the clustering of the metabolic disorders. Dyslipidemia, obesity, disorders of purine metabolism and insulin resistance may be important risk factors for higher SF levels in the elderly.

SF is the major storage form, and an important source of iron in the body. It exists in two states; appoferritin (non-iron state) and the carrying state (containing Fe^3+^ ions). The ability of SF to bind and release iron allows it to maintain stability of hemoglobin iron supply, meaning that levels of iron remain regulated *in vivo*. Iron *in vivo* exists in a variety of forms under a dynamic balance, and iron overload is associated with many diseases. A number of studies have revealed that cardiovascular risk factors, such as dyslipidemia, hypertension, obesity, fasting BG, increased insulin and body iron stores are correlated. These include a study by Williams *et al*, which identified that TG levels, high density lipoprotein levels, BMI anomalies and SF levels (8) were closely correlated. Piperno *et al* revealed that the SF concentration in patients with hypertension was significantly higher than that in the normal population (9), which has been confirmed by further study (10,11). The Deng *et al* study identified a close relationship between SF and blood uric acid (12). Disorders of lipid metabolism, obesity, hypertension, hyperuricemia and abnormal glucose metabolism are pathological states that are often aggregated in the elderly and are major constituents of MS; therefore, elderly individuals with more than one type of metabolic disorder are most likely to exhibit abnormal SF levels. A number of studies have suggested that SF is a risk factor for MS. A study by Bozzini *et al* demonstrated that higher SF levels increased the prevalence of MS (13). The study revealed that with the increase in metabolic disorders in elderly individuals, levels of SF, serum glucose and the insulin resistance index gradually increased. Furthermore, the SF levels were higher in insulin resistant patients than in insulin sensitive patients, regardless of the loss of function of islet cells in the insulin resistant groups. Following correction for confounding variables, other MS-associated factors verified to be independently correlated with SF levels were BMI, TC, TG, SUA and HOMA-IR.

A number of hypotheses for how elevated SF causes metabolic disorders may be considered. i) Iron is able to catalyze lipid peroxidation *in vivo,* producing numerous free radicals that induce body tissue injury (3,4). This results in the functioning of pancreatic islet cells becoming impaired leading to insulin resistance. The oral glucose tolerance test (OGTT) demonstrated that in patients with hemochromatosis, iron overload may cause the liver to develop insulin uptake and utilization disorders, thereby inducing hyperinsulinemia (4). One study indicated that excessive iron deposition in pancreatic β-cells directly affects insulin secretion (5). Another study revealed that bleeding may reduce insulin resistance by reducing iron load (14). Thus, the overload of iron may be influenced by hepatic uptake and utilization of insulin, which may cause insulin resistance (5,9), further confirming that iron proteins may cause or exacerbate insulin resistance. The central aspect of MS is insulin resistance, while iron proteins are involved in the pathogenesis of MS. ii) A high iron load may induce and aggravate disorders of glucose metabolism. Conversely, high blood glucose may have a long-term effect on iron metabolism and iron load. Thus, the roles of iron and glucose metabolism may be bidirectional, acting on each other. This may play a key role in oxidative stress (15). ii) The close association between iron protein and inflammation has been confirmed by many studies and numerous scholars consider that ferritin may be an inflammatory factor (5,9,14,15). One such study has demonstrated this in the long-term process of chronic low-grade inflammation of MS patients (16). Obesity is an important component of MS and is one of its main risk factors due to the close relationship between obesity and a variety of metabolic disorders. A large number of epidemiological studies have revealed that obesity and inflammation are two manifestations of MS (15–17). Certain factors, such as C-reactive proteins, also have inflammatory functions and are considered indicators of inflammatory disease activity. These inflammatory factors are able to prevent the release of free iron from tissues, decrease the total iron binding capacity and decrease the protein levels of serum iron and SF (17). Thus, it may be inferred that in metabolic disorders, ferritin and inflammatory reactions act reciprocally to form a vicious spiral that further exacerbates tissue injury and dysfunction, increased insulin resistance, and ultimately lead to the development of metabolic syndrome. iv) A number of studies have demonstrated close correlations of blood uric acid with cardiovascular disease, dyslipidemia and diabetes, and indicated that hyperuricemia is one of the most important risk factors of cardiovascular disease and is a factor and marker of metabolic disease. Numerous scholars consider that hyperuricemia is a predictor of the risk of early type 2 diabetes and is a manifestation of lipid metabolism disorder (9,15,17). Since hyperuricemia is also a characteristic of MS, it is likely to play an important role in tracking the progress of the occurrence of MS. The Deng Xiaowei study also revealed that blood uric acid and SF are closely correlated (12). In addition, SUA is considered to be an inflammatory factor and is involved in the occurrence of MS through the development of a chronic low-grade inflammatory state.

The present study demonstrated that hypertriglyceridemia and hypercholesterolemia caused a significant increase in SF level, even after adjustment for confounding factors. This is consistent with the findings from the study by El-Gebali *et al* (18) and suggests that iron overload has an important effect on triglyceride and cholesterol metabolism. A number of studies have confirmed the close association between SF and lipid metabolism. Iron ions may cause endothelial cell injury by promoting the low density lipoprotein oxidative modification of arterial smooth muscle cells. Modified low density lipoproteins easily adhere to the artery wall, resulting in high cholesterol and other lipid disorders. The cause of elevated SF levels in obese patients may be due to obesity itself, as it is often accompanied by abnormal blood lipid and glucose metabolism. It may also relate to an increase in the synthesis and release of lipids in the blood. However, the exact mechanism is not entirely clear. A study by Zhang *et al* revealed that TC, TG and low density lipoprotein cholesterol levels in children with obesity were significantly higher than those in children of a normal weigh and identified that SF and TG were significantly associated (19). A study by Piperno *et al* suggested that SF levels were higher in males with hypertension than in those without the condition (20). The current study also demonstrated that hypertension is closely associated with the SF level.

In summary, SF and multiple metabolic disorders in the elderly are closely correlated. Obesity, dyslipidemia, hyperuricemia and insulin resistance are independent risk factors for elevated SF levels in elderly patients. SF increases the risk of MS factors; the higher the SF concentration, the higher the metabolic disorder severity and the more frequent the MS incidence rate. The ability to identify SF levels, as well as their timely monitoring, may be helpful in screening elderly patients for early signs of metabolic disorders and play an important role in the early prevention and treatment of MS. Active weight control and the correction of blood lipid and purine metabolic disorders to improve insulin resistance contributes to preventing cardiovascular and cerebrovascular disease, prolonging life expectancy and improving quality of life for the elderly. Whether reducing iron protein is able to improve metabolic disorders and allow the early prevention of cardiovascular and cerebrovascular diseases has not yet been fully demonstrated and pends further study.

## Figures and Tables

**Table I:** Comparison of the clinical indices of each group.

Patient parameters	MS0 group	MS1 group	MS2 group	MS3 group	F-value	P-value
Cases (male/female)	1150 (650/500)	1250 (750/500)	150 (70/80)	50 (30/20)	-	-
Ages (years)	68.60±5.53	68.48±5.28	67.98±5.68	67.98±5.29	2.17	>0.05
BMI (kg/m^2^)	22.01±2.01	23.91±3.01^a^	24.01±3.01^a^	27.02±3.08^a^,^b^	118.35	<0.01
SUA (μmol/l)	315±45.23	333±47.03^a^	353±50.63^a^,^b^	372±49.23^a^_–_c	17.38	<0.01
LnFINS (mU/l)	1.60±0.5	1.92±0.6^a^	1.94±0.7^a^	2.24±0.7^a^,^b^	92.80	<0.01
Body fat content (%)	31±5	35±6^a^	36±5^a^	38±6^a^,^b^,^c^	156.80	<0.01
LnQUICKI	−0.95±0.11	−1.04±0.08^a^	−1.03±0.10^a^	−1.01±0.09^a^–^c^	91.79	<0.01
LnDI	4.3±0.6	4.0±0.5^a^	4.0±0.6	3.9±0.4	11.01	<0.01
LnHOMA-IR	0.06±0.56	0.42±0.49^a^	0.43±0.78^a^	0.68±0.45^a^,^b^	95.28	<0.01
LnHOMA-β	4.1±0.8	4.4±0.7^a^	4.5±0.6^a^	4.6±0.7^a^	35.31	<0.01
LnUACR	2.6±1.3	3.1±1.1^a^	3.0±1.1^a^,^b^	3.4±1.7^a^,^b^	13.79	<0.01
LnSF	5.2±0.7	5.4±0.7^a^	5.4±0.5^a^	5.6±0.7^a^–^c^	11.38	<0.01

MS0 group, no metabolic disorders; MS1 group, one metabolic disorder; MS2 group, two metabolic disorders; MS3 group, three or more metabolic disorders; Ln, natural logarithm; SUA, serum uric acid; FINS, fasting insulin; QUICKI, quantitative insulin sensitivity index; DI, insulin disposition index; HOMA-IR, homeostatic model assessment-insulin resistance; HOMA-β, homeostasic model assessment-pancreatic β-cell function; UACR, urinary albumin/creatinine ratio; SF, serum ferritin.

a P<0.01, compared with the MS0 group;

b P<0.01, compared with the MS1 group;

c P<0.01, compared with the MS2 group.

**Table II:** Results of stepwise regression analysis between serum ferritin and metabolism.

Factor	B-value	SE	β	P-value
Constant	112.01	90.61		>0.05
Triglyceride	0.69	0.018	0.141	<0.0001
HOMA-IR	0.21	0.020	0.102	<0.0001
Body mass index	0.09	0.003	0.034	<0.0001
Total cholesterol	0.18	0.061	0.081	<0.001
Serum uric acid	0.20	0.072	0.068	<0.001

HOMA-IR, insulin resistance index; B, regression coefficient; SE, standard error; β, standardized regression coefficient.
